# Feasibility and safety of cCD20 RNA CAR-bearing T cell therapy for the treatment of canine B cell malignancies

**DOI:** 10.1186/2051-1426-3-S2-P123

**Published:** 2015-11-04

**Authors:** Jenessa B Smith, M Kazim Panjwani, Keith Schutsky, Josephine Gnanandarajah, Sondra Calhoun, Laurence Cooper, Nicola Mason, Daniel J Powell

**Affiliations:** 1 University of Pennsylvania, Philadelphia, PA, USA; 2 MD Anderson Cancer Center, Houston, TX, USA

## 

CD19 chimeric antigen receptor (CAR) T cell therapy has shown great promise in treating human hematological malignancies. However, the full potential of CAR technology has not been reached in solid malignancies and limitations exist in utilizing genetically identical murine models to interrogate the safety and efficacy of anti-tumor immune therapies. Canines are an outbred population that naturally develop spontaneous disease. Here we have established a treatment model for CAR T cell therapy in outbred dogs with spontaneous B cell lymphoma.

We developed a protocol to isolate, activate, and express CAR in canine T cells. We characterized a methodology utilizing anti-cCD3 antibody with a K562 antigen-presenting cell (APC) line that expresses human CD32 and canine CD86 to activate canine lymphocytes. This method yields robust (50-120x) expansion of cCD4+ or cCD8+ T cells from normal or lymphoma-diseased dogs.

Canine T cells were then engineered utilizing mRNA electroporation to express a CAR targeting the B cell antigen CD20. An anti-canine CD20 (cCD20) CAR was selected for proof of principle studies to establish feasibility of efficient CAR expression and redirected effector function in canine T cells from a healthy and lymphoma-diseased dogs. The cCD20-Z CAR construct was efficiently, but transiently, expressed in canine T cells after mRNA electroporation. cCD20 CAR T cells exhibited specific lysis and IFN-γ secretion in response to cCD20+ target cells. These data indicate successful establishment of a method to isolate, activate, and express a fully functional CAR in canine T cells for use in adoptive immunotherapy. These results rationalized testing of this CAR approach in canines with advanced CD20+ malignancies.

In an IACUC approved protocol, we administered three doses of autologous cCD20-Z RNA CAR+ T cells to a companion canine with advanced B cell lymphoma. Treatment was well-tolerated and no evidence of serious adverse events was observed. The anti-tumor effect, however, was transient, suggesting a need for product optimization. Future study will establish a lenti- or retroviral-based method to modify canine T cells to promote T cell persistence and durable anti-tumor effects *in vivo*.

In conclusion, our study establishes the methodologies and feasibility of anti-cCD20 CAR therapy in canines with spontaneous B cell lymphoma that may represent a future clinical option for such companion pets. This work also lays the foundation for study of a large, outbred animal model that better resembles spontaneous, human malignancies to allow for improved assessment for potential toxicity risks prior to translation into human studies.

